# Small Interfering RNA for LDL-Cholesterol Reduction: A Systematic Review and Meta-Analysis of Randomized Controlled Trials

**DOI:** 10.1016/j.jacadv.2026.102932

**Published:** 2026-07-22

**Authors:** Fellipe Batista, Isadora Mamede, Maria Dacoregio

**Affiliations:** a Department of Innovation, Research and Education, Centro Edson Bueno - CETEB, Rede Total Care, Rio de Janeiro, Rio de Janeiro, Brazil; b Department of Medicine, Federal University of São João del Rei, Divinopolis, Minas Gerais, Brazil; c Department of Medical Genetics, University of São Paulo, São Paulo, São Paulo, Brazil

**Keywords:** hypercholesterolemia, inclisiran, LDL-C, siRNA

## Abstract

**Background:**

Elevated low-density lipoprotein cholesterol (LDL-C) is a major modifiable risk factor for atherosclerotic cardiovascular disease. Although statins remain first-line therapy, many patients fail to achieve guideline-recommended LDL-C targets. Inclisiran, a small interfering RNA that inhibits hepatic proprotein convertase subtilisin/kexin type 9 synthesis, has emerged as a long-acting lipid-lowering strategy. This meta-analysis evaluated the efficacy of inclisiran vs placebo in reducing LDL-C levels.

**Objectives:**

The purpose of this study was to systematically evaluate the efficacy of inclisiran compared with placebo in reducing LDL-C levels across diverse patient populations by synthesizing data from randomized controlled trials and to assess the consistency of LDL-C reduction and safety outcomes in the context of background lipid-lowering therapies.

**Methods:**

PubMed, Embase, and Cochrane Central were systematically searched for randomized controlled trials comparing inclisiran with placebo. The primary outcome was the mean percent change in LDL-C from baseline. A random-effects model using the generic inverse variance method was applied. Heterogeneity was quantified with the I^2^ statistic.

**Results:**

Nine randomized control trials comprising 6,355 participants were included. Inclisiran produced a pooled mean LDL-C reduction of −48.77% (95% CI: −54.26 to −43.27, I^2^ = 95.2%; *P* < 0.001). Follow-up ranged from 0.5 to 1.5 years. Significant heterogeneity was observed (I^2^ = 95%), largely attributable to differences in background lipid-lowering therapy and treatment intensification.

**Conclusions:**

Inclisiran achieves substantial reductions in LDL-C across diverse patient populations. Its twice-yearly dosing schedule and small interfering RNA mechanism of action position inclisiran as a promising long-term lipid-lowering therapy, with potential for broader implementation as clinical experience and outcomes data continue to evolve. (Small Interfering RNA Therapy for LDL-Cholesterol Reduction: A Systematic Review and Meta-Analysis of Randomized Controlled Trials; CRD420251178991.)

## Central Illustration

**Figure:**
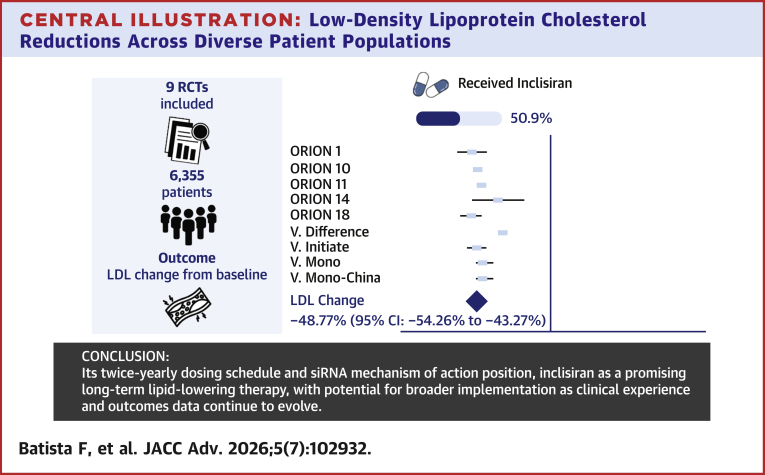

Elevated low-density lipoprotein cholesterol (LDL-C) is a major causal factor in atherosclerotic cardiovascular disease (ASCVD).^1^ While statins remain first-line therapy, many patients do not achieve guideline-recommended LDL-C targets due to inadequate response, intolerance, or high residual risk.^2^ Novel lipid-lowering therapies are therefore essential. Inclisiran is a synthetic small interfering RNA (siRNA) that targets hepatic production of proprotein convertase subtilisin/kexin type 9 (PCSK9), leading to increased LDL receptor expression and substantial LDL-C reduction. Its twice-yearly dosing offers a unique therapeutic approach and has been approved by the U.S. Food and Drug Administration as an adjunct to diet and maximally tolerated statin therapy in adults with hypercholesterolemia.^3^

The American College of Cardiology 2022 Consensus also recommends inclisiran may be used in place of PCSK9 Monoclonal antibodies when the patient has poor adherence to this medication and identifies inclisiran as an important nonstatin option, though its impact on major adverse cardiovascular events (MACE) remains under investigation.^4^

Randomized controlled trials, including the VICTORION-Mono and VICTORION-Difference trials, as well as ORION -10, -11, and other Phase III trials, have consistently demonstrated that inclisiran reduces LDL-C compared with placebo in primary and secondary prevention settings.^5^, ^6^, ^7^

To date, no meta-analysis has synthesized data from both the VICTORION and ORION clinical programs. A pooled synthesis is needed to quantify the overall LDL-C-lowering efficacy, evaluate consistency across populations, and assess safety outcomes. This systematic review and meta-analysis seeks to address this knowledge gap.

## Methods

### Systematic review protocol

This systematic review and meta-analysis was conducted in accordance with the recommendations of the Cochrane Handbook for Systematic Reviews of Interventions and the PRISMA (Preferred Reporting Items for Systematic Reviews and Meta-Analyses) guidelines,^8^ where its checklist can be found in the Supplemental Material 10. The study protocol was prospectively registered in the International Prospective Register of Systematic Reviews database under registration number CRD420251178991.

### Search strategy

A comprehensive search was conducted in MEDLINE (via PubMed), Embase, the Cochrane Central Register of Controlled Trials (CENTRAL), and ClinicalTrials.gov, from inception to the most recent search date, from database inception through November 24, 2025. The search strategy combined controlled vocabulary and free-text terms related to “inclisiran,” “siRNA,” “hypercholesterolemia,” “LDL cholesterol,” and “randomized controlled trial,” with language restriction of English. Additionally, we manually screened the reference lists of relevant reviews and included trials to ensure capture of all eligible studies.

The full search algorithms for each database, including all Boolean operators, MeSH terms, free-text terms, and database-specific adaptations, are provided in Supplemental Table 2.

### Eligibility criteria

Trials were eligible for inclusion if they met the following criteria: 1) randomized controlled design with parallel-group allocation; 2) enrolled adults aged 18 years or older with hypercholesterolemia, ASCVD, or ASCVD risk equivalents; 3) compared inclisiran, in any approved dosing regimen, with placebo; and 4) reported quantitative LDL-C outcomes, including percentage or absolute change from baseline. We excluded studies enrolling pediatric populations, those using nonrandomized or observational designs, trials lacking extractable LDL-C outcomes or not employing a placebo comparator. Trials were excluded if they enrolled patients with familial hypercholesterolemia (FH) as the primary or exclusive population, given the recognized pathophysiological and clinical distinctions between FH and general hypercholesterolemia or ASCVD. Trials in which FH patients represented a small minority within a predominantly non-FH ASCVD population were retained. All study designs, settings (inpatient, outpatient, or community), and geographic locations were considered eligible.

### Study selection and data extraction

Two authors independently screened all titles and abstracts for eligibility. Full texts of potentially eligible studies were assessed for inclusion. Discrepancies were resolved by consensus or by consulting a third reviewer. For all included trials, 2 reviewers independently extracted data using a standardized template, collecting information on study design, sample size, follow-up duration, inclusion criteria, baseline participant characteristics, such as country of origin of the randomized control trial, age, sex, LDL-C, non-high-density lipoprotein cholesterol (HDL-C), HDL-C, apolipoprotein B, total cholesterol, triglycerides, PCSK9, use of statin, use of ezetimibe, intervention and comparator details, time points of LDL-C assessment, and all prespecified primary and secondary outcomes. Extracted safety data included the incidence of total adverse events. When available, least-squares mean differences (LSMDs) were converted into mean differences (MDs) using established statistical procedures to facilitate pooled analysis.

### Outcomes

The primary outcome was the percentage change in LDL-C from baseline at the prespecified follow-up time points used by the original trials (eg, days 90, 150, 360, and 510). Secondary outcomes included absolute changes in LDL-C, the proportion of participants achieving prespecified LDL-C thresholds. We assessed the incidence of treatment-emergent adverse effects and treatment-related adverse effects to evaluate safety.

### Quality assessment

Risk of bias for each included study was assessed independently by 2 reviewers using the Cochrane Risk of Bias 2.0 (RoB 2.0) tool, which evaluates 5 methodological domains: the randomization process, deviations from intended interventions, missing outcome data, measurement of outcomes, and selective reporting.^9^ Discrepancies were resolved through consensus. Risk-of-bias judgments were summarized narratively and visually using the robvis tool.

A funnel plot was generated to assess publication bias, using Egger's tests, and can be found in the Supplemental Appendix (Supplemental Figure 9).^9^

### Statistical analysis

Data were synthesized using a random-effects model with the generic inverse variance method, acknowledging expected clinical and methodological heterogeneity across trials. Between-study variance (τ^2^) was estimated using the restricted maximum likelihood estimator. To account for the small number of included studies (k = 9) and the known tendency of standard random-effects models to produce CIs that are too narrow in this setting, we applied the Hartung-Knapp-Sidik-Jonkman correction to all CIs and *P* values. Continuous outcomes were pooled as MDs with corresponding 95% CIs. Heterogeneity was quantified using the I^2^ statistic and τ^2^. Given the high heterogeneity observed, 95% prediction intervals are reported alongside all pooled estimates to convey the expected range of true effects across a hypothetical new population.

Of the 9 included trials, 7 reported treatment effects as LSMDs (ORION-1, ORION-14, ORION-18, VICTORION-Difference, VICTORION-Initiate, and VICTORION-Mono). The remaining 2 trials—ORION-10 and ORION-11—reported between-group percentage differences, which were entered directly into the pooled analysis as MDs without conversion. VICTORION-Mono China was the sole trial to report a placebo-adjusted percentage change rather than an LSMD or a between-group percentage difference, and was likewise entered directly without conversion.

Exploratory meta-regression analyses were conducted to identify potential sources of heterogeneity, examining statin use (%), follow-up, and baseline LDL-C in the inclisiran arm as continuous moderators (Supplemental Figures 3 to 6). A leave-one-out sensitivity analysis was performed by excluding each trial (Supplemental Figures 7 and 8). A sensitivity analysis was conducted, restricting the analysis to trials with no FH enrollment (Supplemental Figures 1 and 2). Forest plots or bubble plots were generated for all results (Figure 2, Figure 3, Figure 4, Figure 5).

**Figure 1:**
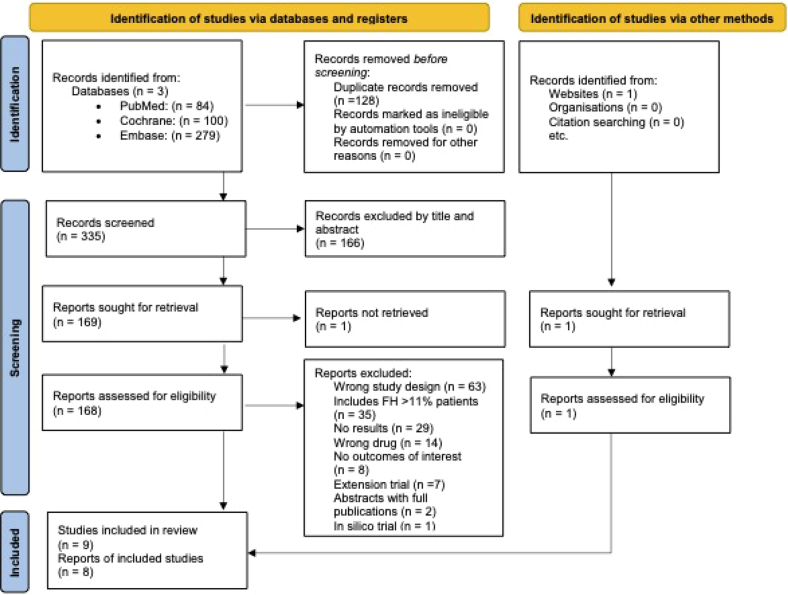
PRISMA Flow Diagram FH = familial hypercholesterolemia; PRISMA = Preferred Reporting Items for Systematic Reviews and Meta-Analyses.

**Figure 2:**
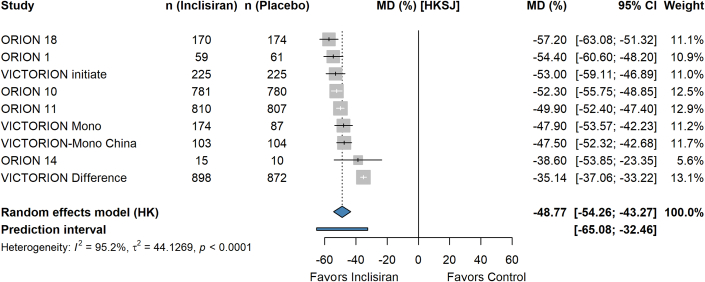
The Mean Difference Percent Change The forest plot comparing the mean difference percent change between inclisiran to placebo regardless of previous or current ioLLT use in both arms. HKSJ = Hartung-Knapp-Sidik-Jonkman; MD = mean difference.

**Figure 3:**
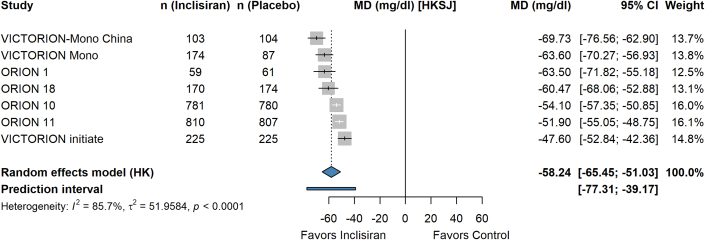
Absolute Low-Density Lipoprotein Cholesterol Mean Difference Change in mg/dL Forest plot analyzing absolute low-density lipoprotein cholesterol mean difference change in mg/dL. Abbreviations as in Figure 2.

**Figure 4:**
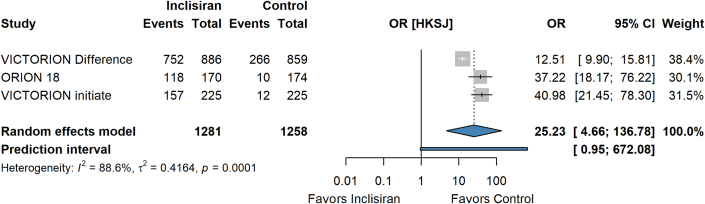
Treatment-Emergent Adverse Event Forest plot of any treatment-emergent adverse event. Abbreviation as in Figure 2.

**Figure 5:**
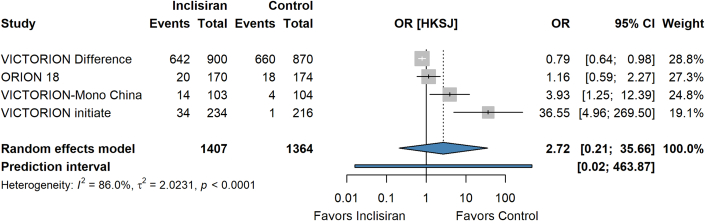
Treatment-Related Adverse Effects Forest plot of treatment-related adverse effects. Abbreviation as in Figure 2.

All analyses were conducted in R v4.5.3 using the meta and metafor packages.

Finally, we assessed the certainty of the evidence for each outcome using the GRADE (Grading of Recommendations, Assessment, Development, and Evaluation) framework. This approach considers risk of bias, inconsistency, indirectness, imprecision, and publication bias to rate the overall quality of evidence as high, moderate, low, or very low.^10^ GRADE summary of findings tables will be provided in the Supplemental Material (Supplemental Table 1).

### Ethical considerations

As this is a systematic review and meta-analysis of previously published studies, no new data involving human participants or animals were collected. Thus, formal ethical approval from an Institutional Review Board/Ethics Committee was not required. All included studies were assumed to have received appropriate ethical oversight as per journal publication standards.

## Results

### Study selection and characteristics

According to the search methodology, 463 studies were extracted from the 3 databases. Following the elimination of duplicate articles and the screening of titles and abstracts, 168 studies were retained; subsequently, upon full-text evaluation, 160 studies were excluded. We included 1 study directly from a website. Nine eligible studies included^5^, ^6^, ^7^^,^^11^, ^12^, ^13^, ^14^, ^15^ 6,355 patients with hyperlipidemia (Figure 1). Baseline characteristics of the included studies are available in Table 1.

**Table 1:** Baseline Characteristics of Studies Used for Analysis

Study, Year	Country	Follow-Up Days	Age^a^, y I/C	Female %	LDL^a^^,^^b^ I/C	Non-HDL^a^^,^^b^ I/C	HDL^a^^,^^b^ I/C	ApoB^a^^,^^b^ I/C	Total Cholesterol^a^^,^^b^ I/C	Triglycerides^a^^,^^b^ I/C	PCSK9^a^^,^^c^ I/C	Use of Statins %, I/C	Use of Ezetimibe %, I/C
ORION 1, 2017 n = 497	Global	180	63/62	39.6	129/126	161/157	48/50	105/103	211/208	127/132	422/417	72/74	NA
ORION 10, 2020 n = 1,56	Global	540	66/65	30.6	104/104	134/134	46/45	94/94	180/180	127/129	422/414	90/89	10/10
ORION 11, 2020 n = 1,617	Global	540	64/64	28	107/103	137/133	49/49	97/95	187/183	135/135	355/353	95/95	6/8
ORION 14, 2023 n = 40	China	90	61/57	32.5	126/133	NA	NA	NA	NA	159/159	345/319	100/100	NA
ORION 18, 2023 n = 344	Asia	360	58/60	25.3	108/109	NA	NA	NA	NA	138/142	458/436	99/97	NA
V China^d^, 2025 n = 207	China	270	48/47	58.5	148/151	177/181	114/115	114/115	226/231	148/152	287/283	0/0	NA
V Difference, 2025 n = 1,770	Global	360	64/64	30.2	84/81	111/109	50/49	85/85	165/162	120/120	NA	100/100	NA
V Initiate, 2024 n = 450	United States	330	65/67	30.9	97/97	127/124	44/47	94/90	171/171	132/119	NA	90/90	0.4/2
V Mono 2025 n = 261	Global	180	45/46	60.1	135/135	163/162	54/54	107/108	217/215	117/116	303/309	0/0	100/100

C = control; I = inclisiran drug; LDL = low-density lipoprotein; PCSK9 = proprotein convertase subtilisin/kexin type 9.

a Mean or median.

b mg/dL.

c ng/mL.

d Conference abstracts.

### Efficacy evaluation

When analyzing cholesterol change, our findings showed that inclisiran lowered the LDL-C in MD –48.77% (95% CI: −54.26% to −43.27%, PI: −65.08 to −32.46, I^2^ = 95.2%) (Figure 2) compared to placebo, regardless of whether both arms were using any type of intensity of background lipid-lowering therapy (ioLLT). Regarding the absolute LDL-C difference, we have observed a MD -58.24 mg/dL (95% CI: -65.45 to −51.03, PI: −77.31 to −39.17, I^2^ = 85.7%) (Figure 3) reduction.

### Safety evaluation

In the safety analysis, no statistically significant differences were observed between the intervention group and the placebo group in the incidence of treatment-emergent adverse effects (Figure 4) or in treatment-related adverse effects (Figure 5).

### Sensitivity analysis

In leave-one-out sensitivity analyses, sequential exclusion of individual trials did not materially change the magnitude or direction of the pooled LDL-C reduction. Meta-regression analyses identified baseline statin use as a significant source of heterogeneity, with higher statin use associated with a smaller incremental LDL-C reduction (slope = 0.14; 95% CI: 0.04-0.24; *P* = 0.043). Baseline LDL-C levels were significantly associated with absolute LDL-C reduction (slope = −0.40; 95% CI: −0.49 to −0.30; *P* = 0.001), but not with percentage change. Follow-up duration was not significantly associated with LDL-C reduction. Despite this, the pooled treatment effect remained large and statistically significant, confirming the robustness of our findings.

A sensitivity analysis restricted to trials with no FH enrollment yielded consistent results for both the percentage change and absolute change in LDL outcomes (Supplemental Figures 1 and 2).

### Quality assessment of the included studies

The quality assessment of the included studies was conducted using the RoB 2 tool, which evaluates potential bias across 5 key domains: 1) bias arising from the randomization process; 2) bias due to deviations from intended interventions; 3) bias due to missing outcome data; 4) bias in measurement of the outcome; and 5) bias in selection of the reported result.

Each domain was assessed independently by 2 reviewers (F.C. and M.D.), with disagreements resolved through discussion or consultation with a third reviewer (S.W.). Studies were classified as having low risk, some concerns, or high risk of bias based on the criteria outlined in the RoB 2 guidance. This systematic approach ensured a rigorous evaluation of study quality and internal validity within the meta-analysis framework.

**Figure 6:**
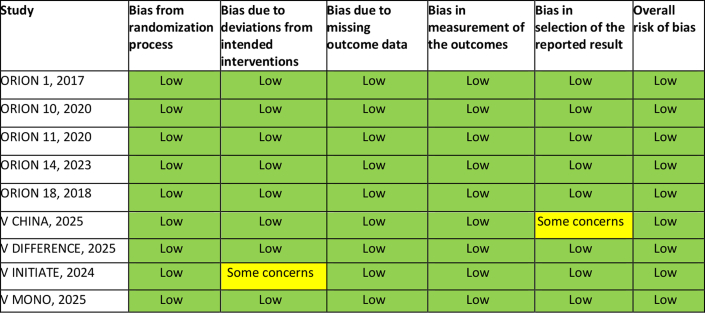
Risk of Bias Summary for Randomized Studies Quality assessment of the studies included in this systematic review and meta-analysis using the Cochrane Risk of Bias 2.0.

In total, 9 studies were evaluated. Of these, all showed an overall low risk of bias, demonstrating consistency across all domains. In contrast, 2 studies raised concerns in at least one domain: The V Initiate (2025) trial due to lack of patient blinding, and the V China (2025) study, which is currently available only as an abstract publication. Despite these concerns, the overall risk of bias was deemed acceptable, supporting the reliability of both the qualitative and quantitative syntheses. A summary of the risk-of-bias assessment across all included studies is presented in Figure 6.

Finally, visual inspection of the funnel plot suggested no substantial asymmetry. Egger’s regression test did not demonstrate significant small-study effects (*P* = 0.118).

## Discussion

This meta-analysis demonstrates that inclisiran significantly reduces LDL-C levels compared with placebo across a wide range of patient populations, regardless of the intensity of background lipid-lowering therapy (ioLLT) (Central Illustration). It can reflect a high generalizability, supporting the external validity of this study.^16^ This consistent LDL-C lowering effect highlights inclisiran’s robust efficacy as a lipid-lowering agent.

**Central Illustration:**
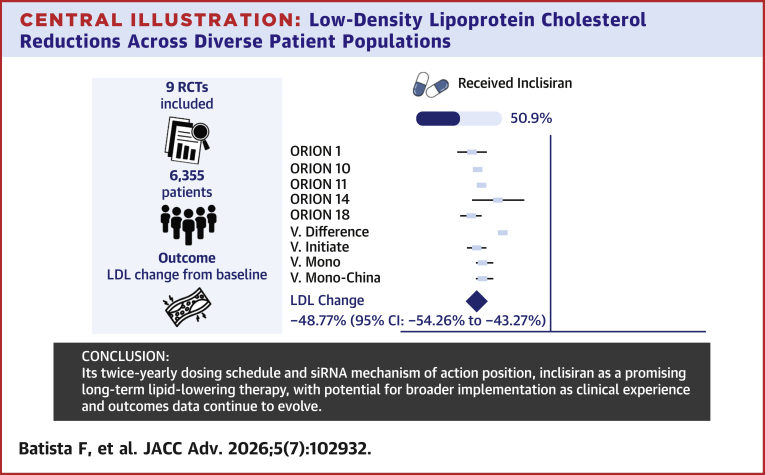
Low-Density Lipoprotein Cholesterol Reductions Across Diverse Patient Populations This Central Illustration summarizes the primary outcome from a systematic review and meta-analysis of 9 randomized control trials (ORION and VICTORION programs) evaluating inclisiran vs placebo or usual care in 6,355 patients with varied profiles: hypercholesterolemia, atherosclerotic cardiovascular disease, statin-naive, non-naive, and optimized lipid-lowering therapy backgrounds. Inclisiran achieved a pooled mean low-density lipoprotein cholesterol reduction of −48.77% (95% CI: −54.26 to −43.27; *P* < 0.001) from baseline, with consistent efficacy independent of baseline therapy or risk level. LDL-C = low-density lipoprotein cholesterol; RCTs = randomized control trials; siRNA = small interfering RNA.

Compared to prior literature, this analysis incorporates, for the first time, data from both the ORION and VICTORION trial programs, providing the most comprehensive synthesis of inclisiran’s efficacy to date. Previous pooled analyses focused on subsets of trials and did not include the latest evidence from the VICTORION studies, making this meta-analysis uniquely informative.

Clinically, inclisiran offers a valuable treatment option for patients who do not achieve LDL-C targets with standard therapies such as statins.^11^ Its twice-yearly dosing schedule may improve adherence, particularly in high-risk populations where sustained LDL-C reduction is critical.^17^

Ongoing cardiovascular outcome trials, including ORION-4, are expected to provide further insight into the long-term cardioprotective benefits of inclisiran.^18^ Emerging Evidence from In Silico Modeling: The SIRIUS in silico trial applied a mechanistic computational model of ASCVD to a large virtual population (n = 204,691), predicting the long-term cardiovascular benefits of inclisiran beyond LDL-C reduction. This innovative approach, calibrated and validated against major outcome trials such as FOURIER^19^ and ORION-10, estimated that inclisiran could reduce the risk of 3-point MACE by 25% over 5 years compared to placebo, when added to high-intensity statins with or without ezetimibe. These findings provide early, model-based evidence supporting the potential cardioprotective effects of inclisiran, several years before results from ongoing outcome trials such as ORION-4 and VICTORION-2P are available.^20^ The use of in silico technology in SIRIUS represents a novel and promising tool for predicting clinical outcomes and may complement traditional trial data to guide therapeutic decisions.

Recent data from the VICTORION-INITIATE program further expand on inclisiran’s clinical profile by directly comparing its lipid-lowering efficacy with other antilipidemic therapies, including statins. This head-to-head evidence demonstrates that inclisiran not only significantly reduces LDL-C compared to placebo but also provides superior LDL-C lowering compared to standard statin therapy in certain patient populations. These findings reinforce inclisiran’s role as an effective alternative or adjunct to conventional lipid-lowering treatments, particularly for patients inadequately controlled by statins alone. This comparative advantage, combined with inclisiran’s biannual dosing regimen, supports its potential to improve both lipid management and treatment adherence in clinical practice.^12^

VICTORION Difference program further substantiates inclisiran’s potent LDL-C lowering effect, demonstrating superiority over placebo even when both arms receive intensive background lipid-lowering therapies, including high-intensity statins and ezetimibe. This confirms inclisiran’s additive efficacy beyond standard lipid-lowering regimens and highlights its potential as a valuable treatment option for patients who remain above LDL-C targets despite optimized therapy.^6^

The substantial heterogeneity observed across studies appears to be partially explained by differences in background statin therapy. Studies with higher statin use demonstrated smaller incremental LDL-C reductions with inclisiran, likely reflecting overlapping mechanisms of PCSK9 suppression.^21^ Additionally, baseline LDL-C influenced absolute but not percentage reductions, consistent with expected biological scaling effects. Other factors, including follow-up duration, did not significantly contribute to between-study variability. The absence of significant asymmetry on funnel plot inspection and a nonsignificant Egger’s test suggests a low likelihood of publication bias, supporting the robustness of the findings.

In the VICTORION-Mono trial, which enrolled statin-naive patients initiating inclisiran, significant LDL-C reductions were observed, demonstrating inclisiran’s efficacy independent of prior statin exposure. Conversely, ORION-18 included patients not statin-naive but showed similar or even greater percentage LDL-C reductions compared to control populations.

This highlights inclisiran’s versatility as a therapeutic agent effective both as monotherapy and adjunctive treatment, expanding its potential use across diverse patient populations with varying background lipid-lowering regimens.

Recent work suggests that inclisiran is potentially a cost-effective agent for the treatment of elevated LDL-C in patients with severe dyslipidemia.^22^^,^^23^ A recent study from Spain acknowledged that achievement of low LDL-C thresholds after hospitalization for ACS reduced overall health care expenditures from 7,000 Euros/y to around 5,000 euros/y including fewer days in hospital, fewer subsequent ED visits, and improved health overall.^22^ Data from the work with inclisiran have demonstrated an excellent likelihood of achieving LDL-C thresholds of <70 mg/dL and <55 mg/dL in at least 75% and 66% of patients, respectively.^24^ The Institute for Clinical and Economic Reviews Midwest Comparative Effectiveness Public Advisory Council also suggested that inclisiran meets appropriate cost-effectiveness thresholds and adds favorably to the cost-benefit analysis of PCSK9 therapy when combined with statin treatment. The work acknowledged that inclisiran is more expensive than ezetimibe but was also at substantially more effective at LDL-C lowering, thus offsetting the additional incremental costs with a potential cost savings benefit because of the potential for reductions in MACE from LDL-C lowering. Taken together, these papers suggest that inclisiran is a potent LDL-C lowering therapy that has the potential of significant cost savings in the post-MI population and is cost-effective in the population with FH.

Strengths of this meta-analysis include the integration of ORION and VICTORION trial data, rigorous adherence to PRISMA guidelines, and thorough assessments of risk of bias and evidence quality using GRADE methodology. All of the studies we cite required that patients be treated with maximally tolerated statin therapy or have documented statin-intolerance. The use of background lipid-lowering therapy was consistent across all of the studies with over 90% of patients on background statin agents and with nearly 70% on high-intensity statins with one exception. The exception was the Victorian mono (V-Mono) trial which included patients who did not meet guideline-directed indications for use of statins. The patients in V-Mono were randomized to inclisiran, ezetimibe or placebo, and approximately 25% were treated with inclisiran.

### Study Limitations

Limitations must be acknowledged: the lack of individual patient-level data restricts detailed subgroup analyses; variability in background statin therapy across trials may introduce heterogeneity, and the current scarcity of long-term cardiovascular outcome data limits conclusions about sustained clinical benefit.

## Conclusions

This meta-analysis confirms that inclisiran significantly reduces LDL-C levels compared with placebo across diverse populations, independent of background lipid-lowering therapy intensity. By integrating data from both ORION and VICTORION trials, it provides a comprehensive evaluation of inclisiran’s efficacy to date. Head-to-head comparisons show superior LDL-C reduction vs statins, highlighting inclisiran’s value as an adjunct or alternative in lipid management. Overall, inclisiran is a robust, adherence-friendly lipid-lowering therapy with demonstrated efficacy and promising cardiovascular benefits, with potential for broader implementation as clinical experience and outcomes data continue to evolve

Perspectives **COMPETENCY IN MEDICAL KNOWLEDGE:** Inclisiran, a siRNA targeting hepatic PCSK9 synthesis, achieves a mean LDL-C reduction of approximately 49% compared with placebo across diverse patient populations, independent of background lipid-lowering therapy intensity. This siRNA-based mechanism provides sustained LDL-C lowering with twice-yearly dosing, offering a distinct approach from PCSK9 monoclonal antibodies, ezetimibe, and statins. Patient Care: Inclisiran represents a valuable option for patients not achieving guideline-recommended LDL-C targets despite maximally tolerated statin therapy. Its twice-yearly subcutaneous regimen may improve adherence compared with daily oral therapies or frequent self-injected treatments, particularly in high-risk populations where sustained LDL-C reduction is critical for cardiovascular risk reduction. Systems-Based Practice: Implementation requires consideration of in-office administration models every 6 months. Value-based prescribing should account for drug acquisition costs alongside potential downstream savings from prevented cardiovascular events through improved adherence and LDL-C goal attainment.

**TRANSLATIONAL OUTLOOK:** Translating biomedical research from clinical trials to individual patient care can expedite new therapies through multidisciplinary collaboration. Effective translational medicine facilitates implementation of evolving strategies for prevention and treatment of disease in the community. The National Institutes of Health has recognized the importance of translational biomedical research, emphasizing multifunctional collaborations between researchers and clinicians to leverage new technology and accelerate the delivery of new therapies to patients. Ongoing cardiovascular outcomes trials (ORION-4, VICTORION-2P) will provide definitive evidence on MACE reduction. Future research should focus on real-world effectiveness studies comparing inclisiran with ezetimibe and PCSK9 monoclonal antibodies, cost-effectiveness analyses incorporating adherence patterns, and identification of patient subgroups most likely to benefit from this siRNA-based therapeutic approach..

## Funding support and author disclosures

This meta-analysis will receive grant support from Instituto de Ensino, Pesquisa e Gestão em Saúde–IEPEGES and 10.13039/100000871Mayo Clinic. The authors have reported that they have no relationships relevant to the contents of this paper to disclose.
